# 2784. Rates of Susceptibility and Heteroresistance to Novel Antibiotic Combinations in Carbapenem-Resistant Enterobacterales Isolates - Emory Healthcare, 2016-2021

**DOI:** 10.1093/ofid/ofad500.2395

**Published:** 2023-11-27

**Authors:** Christina K Lin, Alex M Page, Sarah Lohsen, Ahmed Babiker, Jesse T Jacob, Sarah W Satola, Jessica Howard-Anderson

**Affiliations:** Emory School of Medicine, Atlanta, Georgia; Emory University School of Medicine, Atlanta, Georgia; Emory University School of Medicine, Atlanta, Georgia; Emory University School of Medicine, Atlanta, Georgia; Emory University School of Medicine, Atlanta, Georgia; Emory University School of Medicine, Division of Infectious Diseases, Atlanta, Georgia; Emory University, Atlanta, Georgia

## Abstract

**Background:**

CRE infections are frequently treated with new beta-lactam/beta-lactamase inhibitors (BL/BLI): imipenem-relebactam (I-R), ceftazidime-avibactam (CZA), and meropenem-vaborbactam (MVB). However, the frequency of susceptibility and heteroresistance (HR), defined as the presence of an antibiotic-resistant subpopulation within a primary susceptible population, to these newer antibiotics is unknown. Here we evaluate rates of susceptibility and HR to newer BL/BLI in a convenience sample of clinical CRE isolates as potential reasons for clinical failure.

**Methods:**

We created an antibiogram for carbapenem-resistant *Enterobacter cloacae*, *Escherichia coli* and *Klebsiella pneumoniae* isolates collected between 2016–2021 as part of routine clinical care from two >500-bed academic hospitals. We tested the first isolate per patient for susceptibility to I-R, CZA, and MVB by broth microdilution. We used population analysis profiling (PAP) to test for HR to CZA and I-R.

**Results:**

Of 328 CRE isolates, 74 (22%) were from sterile sites, 151 (46%) from urine, 43 (13%) from the respiratory tract, and 60 (18%) from other non-sterile sites. For *E. cloacae* (n=152), 93% were susceptible to I-R, 98% to CZA, and 99% to MVB. For *E. coli* (n=53), 89% were susceptible to I-R, 94% to CZA, and 93% to MVB. For *K. pneumoniae* (n=123), 90% were susceptible to I-R, 94% to CZA, and 93% to MVB (Table 1). Over time, decreases in the activity of all 3 BL/BLI were noted. Compared to 2016, the percentage of susceptible CRE isolates decreased by 11% (98% to 87%) for I-R, 7% (98% to 91%) for CZA, and 8% (98% to 90%) for MVB (Figure 1). The percentage of isolates with HR to CZA was 7.2% for *E. cloacae*, 1.9% for *E. coli*, and 4.9% for *K. pneumoniae*. For I-R, the percentage of HR isolates was 4.1% for *E. cloacae*, 9.6% for *E. coli*, and 10.3% for *K. pneumoniae*.

**Table 1.**
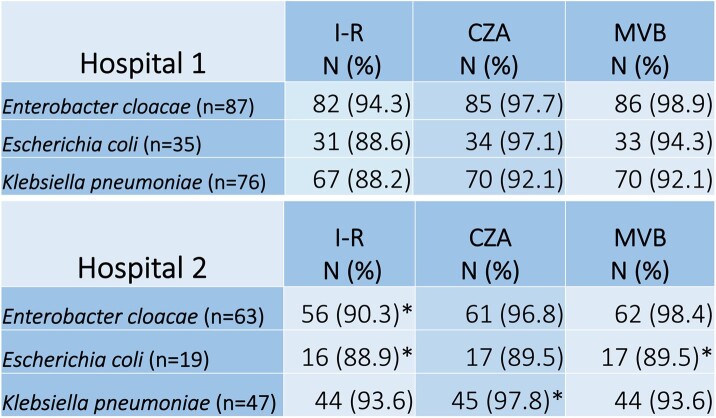
Proportion of carbapenem-resistant Enterobacterales isolates susceptible to new beta-lactam/beta-lactamase inhibitors in two academic hospitals. N = number of antibiotic susceptible isolates, followed by percentage (%) of susceptible isolates. Asterisk (*) indicates if not all isolates were tested against the specified antibiotic. I-R = imipenem-relebactam CZA = ceftazidime-avibactam MVB = meropenem-vaborbactam

**Figure:**
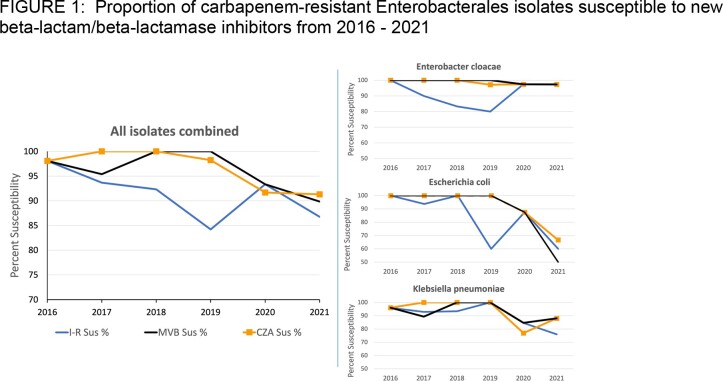

The percent susceptibility of carbapenem-resistant Enterobacterales isolates to the specified antibiotic was graphed per year for all isolates and by individual species. The isolates from two academic hospitals were combined. I-R Sus % = Percent susceptibility to imipenem-relebactam MVB Sus % = Percent susceptibility to meropenem-vaborbactam CZA Sus % = Percent susceptibility to ceftazidime-avibactam

**Conclusion:**

In our study on the activity of newer BL/BLI against CRE, susceptibility remains high and HR is rare. While newer BL/BLIs remain highly active against CRE, the observed decrease in susceptibility over time suggests a need for ongoing antibiotic stewardship.

**Disclosures:**

**Ahmed Babiker, MBBS**, Roche: Advisor/Consultant

